# Editorial: Myeloid-derived suppressor cells in health and diseases: harnessing their roles in the development of new immunotherapy approaches

**DOI:** 10.3389/fimmu.2024.1411391

**Published:** 2024-06-05

**Authors:** Sergej Tomić, Shengjun Wang, Miodrag Čolić

**Affiliations:** ^1^ Institute for the Application of Nuclear Energy, University of Belgrade, Belgrade, Serbia; ^2^ Department of Immunology, Jiangsu Key Laboratory of Laboratory Medicine, School of Medicine, Jiangsu University, Zhenjiang, China; ^3^ Department of Laboratory Medicine, Affiliated Hospital, Jiangsu University, Zhenjiang, China; ^4^ Department of Medical Sciences, Serbian Academy of Sciences and Arts, Belgrade, Serbia

**Keywords:** myeloid-derived suppressor cells, cancer, autoimmunity, pregnancy, ageing, natural products

Myeloid-derived suppressor cells (MDSCs) are a heterogeneous cell population with critical roles in the regulation of immune response. Initial research designated MDSCs as pathologically activated myeloid cells with potent immunosuppressive activities (1). However, additional roles of MDSCs in the regulation of immune response were demonstrated besides immunosuppression (1, 2). Emergency myelopoiesis induced by chronic inflammation is the main generator of MDSCs. Major subsets of MDSCs are polymorphonuclear (PMN)-MDSCs and mononuclear (M)-MDSCs, with specific phenotypes described in both mice and humans (1, 3). An additional subtype in humans designated as early (e)MDSCs was described, according to the lack of specific granulocytic or monocytic markers (3). Moreover, in many pathological conditions, different myeloid cells may acquire MDSCs-like properties (4), further contributing to MDSCs heterogenicity and complexity. Although primarily implicated in pathological conditions, such as cancer, infection, autoimmunity and obesity, emerging facts indicate MDSCs involvement in physiological conditions such as pregnancy, neonates, and ageing (5). These data illustrate the complexity of MDSCs development, regulation, and functions, so their roles in many conditions are still insufficiently investigated (1, 2, 5). The Research Topic entitled: *Myeloid-Derived Suppressor Cells in Health and Diseases: Harnessing Their Roles in the Development of New Immunotherapy Approaches* gathered 5 exquisite papers from totally 36 authors, illuminating the multifaceted roles of MDSCs in oncogenesis, maternal-fetal interface and ageing, all of which collectively deepen our understanding of MDSCs biology and opened new insight for the potential development of more efficient immunotherapeutic approaches.

Wang et al. critically discussed about the role of pattern recognition receptors (PRRs) in MDSCs functions in the context of tumorigenesis. These receptors recognize pathogen- and damage-associated molecular patterns (PAMPs and DAMPs, respectively) triggering different signaling pathways that regulate expansion, survival, functions and differentiation of MDSCs. Through meticulous overview of PRR agonist effects on MDSCs subtypes, the authors emphasized those with a high anti-tumor potential according to their capacity to reduce MDSCs frequency and suppressive functions (i.e. endosomal toll-like receptors, TLRs), as well as those which display pro-tumor effects through promotion of MDSCs expansion, survival, accumulation in cancer (i.e. TLR4). These authors emphasized the importance of further deciphering the roles of PRRs agonists and their combinations on MDSCs, as a promising strategy to revert suppressive myeloid cells to an antitumor phenotype for more effective cancer immunotherapy.

Some viruses have exploited PRRs for establishing persistent infections, which can cause tumorigenesis. Glover et al. critically discussed about known and potential roles of MDSCs subsets in immune evasion by oncogenic viruses, including human gamma herpesviruses, human papillomaviruses, hepatitis viruses, human T cell leukemia virus, and Merkel cell polyomavirus. Each of these viruses perturb numerous cellular pathways, wherein MDSCs were identified as important factors. Along with viral-specific mechanisms of immune evasion and oncogenesis, the authors indicated that IL-6, TGF-β and GM-CSF are common factors produced by the infected cells, which drives emergency myelopoiesis and MDSCs expansion. Moreover, some factors such as ER stress were indicated as important for PMN-MDSCs generation, whereas TLR stimulation and TGF-β were designated as more important for M-MDSCs. These authors also emphasized that further studies on MDSCs in viral-induced tumors could lead to development of innovative immunotherapies for these diseases.

Besides cancer, MDSCs and the molecular mechanisms underlying their functions were envisioned as excellent therapeutic targets in reproductive medicine. Pang et al. extensively reviewed the emerging data on the interaction between MDSCs and key cells of maternal-fetal interface (i.e. T cells, NK cells, trophoblasts, etc.) that control the processes from embryo implantation to postpartum. The data from human and animal studies indicated clearly that MDSCs deficiency leads to spontaneous miscarriage and pregnancy complications. The regulation of MDSCs functions by hormones, and MDSCs effects on angiogenesis and trophoblasts functions were particularly emphasized as critical dysregulation points, leading to complications such as intrauterine growth restriction and preeclampsia. Besides MDSCs suppressive functions in pregnancy, their pro-inflammatory and protective functions in neonates was indicated as well. Cumulatively, a better understanding of MDSCs roles in maternal-fetal interface could lead to identification of new targets that are able to promote positive reproductive outcomes.

Aging is characterized by an enhanced myelopoiesis and MDSCs accumulation, which positively correlates with increased prevalence of myeloid-driven inflammation, myeloid leukemias, and related pathologies. In an original research paper, Kwack et al. investigated the role of Tristetraprolin (TPP), an RNA-binding protein regulating the translation of immune-related cytokines and chemokines, in age-related myelopoiesis. The downregulation of TTP expression occurring during aging, was modeled in mice by using cutting-edge knockouts for total and myeloid-restricted TTP deficiency. The authors demonstrated that TTP deficiency in myeloid progenitors leads to expansion of M-MDSCs, their increased expression of CCR2 and the serum levels of CCL2. These experiments demonstrated a previously unrecognized role for TTP in regulating age-associated expansion of myeloid progenitors and M-MDSCs, as well as their recruitment to sites of injury and inflammation via CCR2-CCL2 axis.

The targeted modulation of MDSCs is seen as a promising strategy for successful immunotherapy, and various innovative approaches are being developed. In an original research paper, Shen et al. studied the effects of *Angelica sinensis* polysaccharide (APS), a natural immune enhancing agent used in clinics, on MDSCs functions. It was demonstrated that APS expands different lymphoid and myeloid subsets *in vivo*. However, the authors identified that in the presence of GM-CSF, APS can promote the proliferation, differentiation, CD206 expression and immunosuppressive functions of MDSCs through STAT1 and STAT3 signaling pathways. These results emphasize that great care should be taken when applying APS in cancer supplementation therapy, due to potentially adverse effects mediated by MDSCs.

The articles of the Research Topic significantly contributed to a better understand MDSCs’ complex nature oscillating between immune regulation and pathology promotion, particularly in oncology, reproductive medicine and aging, for which we are much grateful to the contributing authors and the excellent reviewers.

## Author contributions

ST: Conceptualization, Writing – original draft, Writing – review & editing. SW: Writing – review & editing. MC: Writing – review & editing.
